# Biomimetic Superhydrophobic Hollowed-Out Pyramid Surface Based on Self-Assembly

**DOI:** 10.3390/ma11050813

**Published:** 2018-05-16

**Authors:** Weipeng Luo, Bin Yu, Dingbang Xiao, Meng Zhang, Xuezhong Wu, Guoxi Li

**Affiliations:** 1 College of Mechatronics Engineering and Automation, National University of Defense Technology, Changsha 410073, China; z.mengdr@gmail.com (M.Z.); xzw@nudt.edu.cn (X.W.); 2 QuantumCTeK Co., Ltd., Hefei 230031, China; xpp105106@126.com

**Keywords:** superhydrophobic, biomimetic, self-assembly, SU-8 photoresist

## Abstract

In this paper, we present a periodic hollowed-out pyramid microstructure with excellent superhydrophobicity. In our approach, T-topping pillars and capillary-induced self-assembly methods were combined with the photolithography process to fabricate a hollowed-out pyramid structure. First, a wideband ultraviolet source without a filter was used to fabricate the T-topping pillars during the exposure process; then, the evaporation-induced assembly collapsed the pillars and formed the hollowed-out pyramid structure. Scanning electron microscopy images showed the microstructures of the prepared surface. The contact angle of the surface was 154°. The surface showed excellent high temperature and ultraviolet irradiation tolerance, and the contact angle of the surface barely changed when the temperature dropped. This excellent environmental durability of our superhydrophobic surface has potential applications for self-cleaning and friction drag reduction under water.

## 1. Introduction

Friction drag is an important factor for marine vehicles. Drag reduction techniques have become a focus to increase vehicle speed and save fuel. Several techniques have been proposed to reduce the friction drag, including water repellent walls [1,2], microbubbles [3,4], surface microstructure [5], and surfactant additives [6].

Bionics and experiments showed that superhydrophobic surfaces have an obvious drag reduction effect due to the ability to retaining microbubbles [1,7,8,9]. The retention of microbubbles on the surfaces can reduce friction drag by flowing slipping. However, the microbubbles disappear rapidly because of dissolution and scouring by water. Increasing the number of resident microbubbles has become an important issue. 

The *Salvinia molesta* leaf has excellent superhydrophobicity and air-retaining properties under water because of its hollowed-out pyramid-like structure [10]. Hence, we focused on fabricating a superhydrophobic surface with a hollowed-out pyramid-like structure. Evaporation-induced assembly of pillars was used to fabricate the structure. A variety of factors that may affect the self-assembly process have been studied at the micrometer scale [11,12,13,14,15,16]. Capillary force was found to be the most important factor. The assembly process was considered as a competition between elasticity and capillarity for wet micropillar systems [17,18]. Capillary force on an individual pillar in a system of four pillars was explored by Chandra [19]. Adhesion force and capillary force are the major factors used to bend the ultra-high aspect ratio silicon (Si) nanowires [20]. 

Adhesion between the micro pillars is another important factor that determines the final assembly [21]. A polymer solution was used to provide an adhesion force between the pillars and to improve the self-assembly stability [22]. Laser printing has been used to make micro pillars, and the collapsed structure was formed by self-assembly [23]. 

Inclined lithography was also used to fabricate sloping micro structures [24,25]. Sloping micro structures have been fabricated using negative thick photoresist such as SU-8 [26,27,28]. Inclined lithography can be used to create the hollowed-out pyramid-like structure, requiring incline lithography in four different directions and an exposure machine to adjust the tilted angle of the stage. However, performing alignment to ensure the adjacent micro pillars join together is difficult.

These studies built a strong foundation for fabricating hollowed-out pyramid-like structures. In this paper, we fabricated a superhydrophobic surface based on the hollowed-out pyramid-like microstructure. Since the discovery of the lotus effect, the preparation of the biomimetic superhydrophobic surfaces has aroused considerable interest among researchers. From observation and preparation, wettability was found to be related to the surface free energy and structure roughness.

Inspired by the plants and animals in nature, scientists fabricate numerous biomimetic superhydrophobic surfaces via various smart materials routes to construct rough surfaces and low-free-energy materials [29,30,31]. The major works focused on creating super-hydrophobic surfaces by modifying low-surface-energy groups, such as fluorinate and fluoroalkyl silaneor [32,33]. Some studies focused on the fabrication and application of hydrophobic membranes [34,35,36]. Non-fluorinated hydrophobic grafting molecules were used to modify hydrophilic ceramic membranes [35,36]. Others have changed the microstructure of the surfaces, including chemical etching and periodic arrays made using the micro-electromechanical systems (MEMS) process. Usually, the two methods are combined to make superhydrophobic surfaces.

Using the periodic array microstructures, superoleophobic and superrepellent surface fabrication has been achieved [37,38]. A T-structure pillar can change the geometric edge angle of the straight wall pillar, which can cause droplet overhang on the T-structure pillar array surface. Additionally, the nanoscale doubly reentrant structure considerably improves the wettability of the surface, which can make the prepared surface of any material superrepellent [38].

The durability of superhydrobic surfaces is another important aspect. Poor durability limits the practical applications. Mechanical abrasion, chemical stability, organic solvents, high temperature. and ultraviolet (UV) irradiation are harsh conditions for superhydrobic surfaces. Many durable superhydrobic surfaces have been fabricated [39,40,41,42,43,44,45], and even self-recovering superhydrophobic coatings have been reported [46,47]. A superhydrophobic cotton fabric with excellent washing durability, solvent resistance, and chemical stability was prepared from an SU-8 derived coating, the water contact angle was 163°, and the sliding angle was 2° [41]. A simple dip coating approach was reported for preparing super durable superhydrophobic materials; the mechanical abrasion, long time immersion in various liquids, and repeated washing had no obvious influence on the superhydrophobicity [44]. Most research focused on fabricating self-cleaning surfaces with superior mechanical abrasion, chemical, and UV resistance, but the effect of temperature changes on the wettability of the surfaces must be researched. 

In this paper, we combined the T-topping structure and capillary-induced self-assembly in the photolithography process. Only the geometry structure of the surface was changed without addition of low surface-energy ingredients. The contact angle of the surface was 154 ± 2°, the sliding angle of the surface was 10 ± 1°. The sample showed excellent wettability after high temperature thermal treatment and ultraviolet irradiation. The contact angle of the surface fluctuated slightly in the cooling process. The interspace surrounded by the hollowed-out pyramid-like structures was better for air retention under water, allowing the surface to have potential for drag reduction under water.

## 2. Materials and Methods 

### 2.1. Materials 

In this study, all chemicals were used as received. The SU-8 2100 Negative Photoresist and SU-8 developer were purchased from Micro Chem (Westborough, MA, US). Other chemicals, including ethanol (C_2_H_5_OH), acetone (C_3_H_6_O), and Isopropyl Alcohol (IPA) were purchased from Sinopharm (Shanghai, China). Silicon (Si) slides with 500 nm thick silicon dioxide were used as the substrate. Ultrapure water obtained from a Millipore Milli-Q system (resistance rate >18.2 MΩ·cm) was used for sample preparation. The mask plate was manufactured by Shenzhen Newway Photomask making Co., Ltd. (Shenzhen, China).

### 2.2. T-Topping Pillar Preparation 

Many researchers improved the fabrication technology to avoid the T-topping effect of SU-8 structure fabrication [48,49,50]. However, the T-topping pillar was the key structure here. Because the contact angle of SU-8 film is just 82°, it is insufficient for straight wall pillars to sustain droplet in the Cassie state. The T-topping pillar allowed the surface to sustain water in the Cassie state [51]. The graphic on the mask plate was an array of circles in two dimensions. The period was 100 µm and the diameter of the circle was 20 µm. The specific fabrication steps were as follows: (1) Ultrasonic cleaner (KQ-800KDB, Kunshan Ultrosonic instrument Co., Ltd., Kunshan, China) was used to wash the silicon wafer. The wafer was washed with acetone, ethanol, and deionized water. The washing time for each solution was 5 min. Then the wafer was dried in the air and cleaned by oxygen plasma. The SU-8 photoresist was heated to 40 °C and maintained at this temperature for 30 min to decrease the viscosity and remove the air bubbles. (2) The SU-8 photoresist was poured on the prepared wafer, and the wafer was spin-coated at 3000 rpm for 20 s using a spinner (EL-S-200TT, Obducat, Radolfzell, Germany). The thickness of the SU-8 film was about 100 µm. Then the sample was prebaked at 65 °C for 10 min to evaporate the organic solvent on the hotplate (KW-4AH, Chemat technology Inc., Northridge, CA, US). Finally, the SU-8 developer was used to decrease the marginal thick part of the SU-8 film. (3) The mask plate was placed upon the wafer with Su-8 film, then the sample was exposed for 30 s to the UV source at an exposure dose of 8 mW/cm^2^. A wideband ultraviolet source without a filter was used in the exposure step to obtain T-topping pillars. (4) The sample was baked at 95 °C for 10 min on the hotplate and developed in the SU-8 developer for 6 min. Then the wafer sample was rinsed in the deionized water. Finally, the prepared sample was dried in the air naturally.

### 2.3. Preparation of Mask Plate for the Hollowed-Out Pyramid-Like Structure

**Figure 1:**
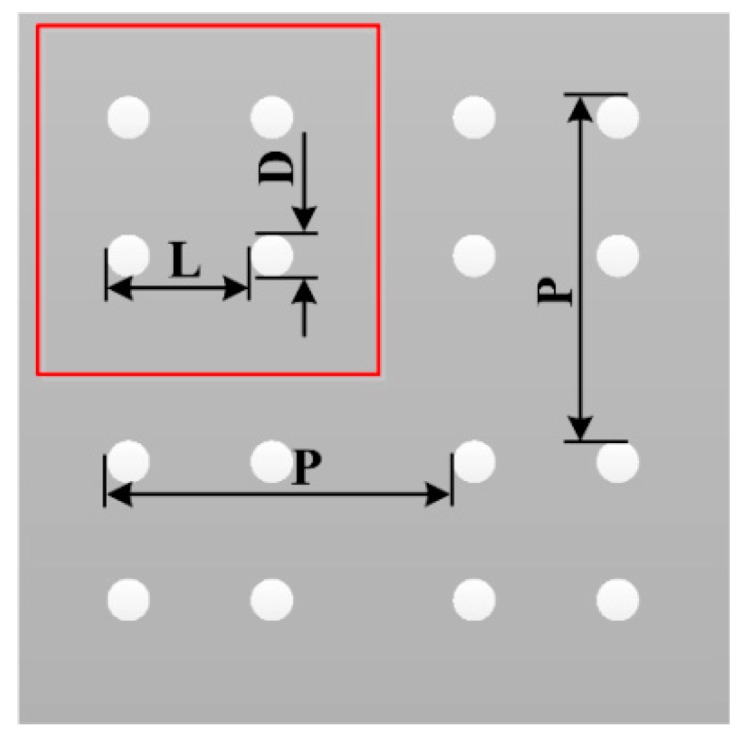
Schematic illustrations of the mask plate. The area surrounded by the red line is the unit of the array. The unit was composed of four adjacent circles. P is the array period (100 µm), L is the distance of the two adjacent circles (50 µm), and D is the diameter of a single circle (10 µm).

The mask graphic was an array of units composed by four adjacent circles in two dimensions. The parameters of the mask graphic were calculated and designed. As shown in Figure 1, the area surrounded by the red line was a repeat unit of the array. The unit was composed of four adjacent circles, P is the array period, L is the distance of the two adjacent circles, and D is the diameter of a single circle. According to the previous studies of hydrophobicity and self-assembly, the parameters of the mask graphic were chosen as: P = 100 µm, L = 50 µm, and D = 10 µm.

### 2.4. Hollowed-Out Pyramid-Like Structure Preparation

**Figure 2:**
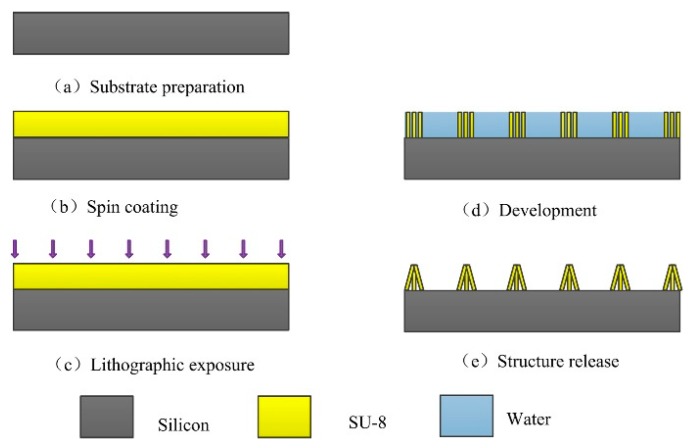
Schematics of the procedures of fabricating superhydrophobic surface with hollowed-out pyramid array structures. (**a**–**e**) The lithography procedures, including substrate preparation, spin-coating, exposure, development, and structure release. (**d**,**e**) The vertical section view that was cut though the center of the hollowed-out pyramid microstructure; three pillars are visible.

All steps were identical to the T-topping pillar preparation. The only difference was the last step of structure release. The details are shown in Figure 2. When the surface sample was removed from water after the development process, the micro pillars had formed, with water filling the space around the pillars. The water surrounded by the four adjacent pillars (in the repeated unit) evaporated more slowly. With the water evaporating, a liquid bridge formed between the four adjacent pillars. With the evaporation of the water, the capillary force made the top of the adjacent pillars join together. The pillars collapsed to the periodic hollowed-out pyramid array structure by self-assembly.

### 2.5. Characterization

The morphologies of the solid phase nanostructures were investigated using field emission scanning electron microscopy (FESEM; Hitachi S-4800, Hitachi Ltd., Tokyo, Japan) at 5 kV. The static contact angles of the sample were measured by the contact angle measurement instrument. Contact angle images were collected with a high-resolution camera, and the tangent lines were drawn using Microsoft Visio 2010 (Microsoft, Redmond, WA, USA). Then, the contact angles were measured using a protractor, with an accuracy of ± 2°. Water droplets of about 7 µL were dropped on the sample from a distance of 0.3 cm by vibrating the pipette. The sliding angles were measured with the assistance of a rotatable platform with an angle scale, with an accuracy of ± 1°.

### 2.6. Temperature Durability Test

The wettability of the sample under harsh environments was tested, including high and low temperatures. In the high temperature durability tests, the sample was 20 × 20 mm^2^. The sample was heated by a hotplate. First, the sample was placed on the hotplate for 1 h at 100 °C; next, the sample was removed and cooled to room temperature; third, the contact angles of the sample were measured and then the sample was placed back on the hotplate for another 1 h at 100 °C. The process was repeated 8 times. Finally the temperature of the hotplate was set to 150 °C, and the experiment above was repeated.

In the cooling tests, the sample was 20 × 20 mm^2^. The relationship between the static contact angles and the temperature was investigated. The sample was cooled by a ZL-04AGT thermostat (ESPEC, Osaka, Japan). The sample was placed on a refrigeration table, and the contact angle measurement instrument was placed in the thermostat to shoot the side view of the water droplet. The temperature fell from 10 to −5 °C, and the cooling rate was 3 °C/min. The humidity was 30%.

### 2.7. UVDurability Test

In the UV durability tests, the UV source (laser power 8 mW/cm^2^, k = 365 nm) of the lithography machine (ABM Inc., San Jose, CA, USA) was used for irradiation. The distance between the UV light source and the samples was about 5 cm. The samples were placed in the UV chamber for up to 90 min and the contact angles of the sample were measured every 10 min.

## 3. Results and Discussion

### 3.1. T-Topping Pillar

**Figure 3:**
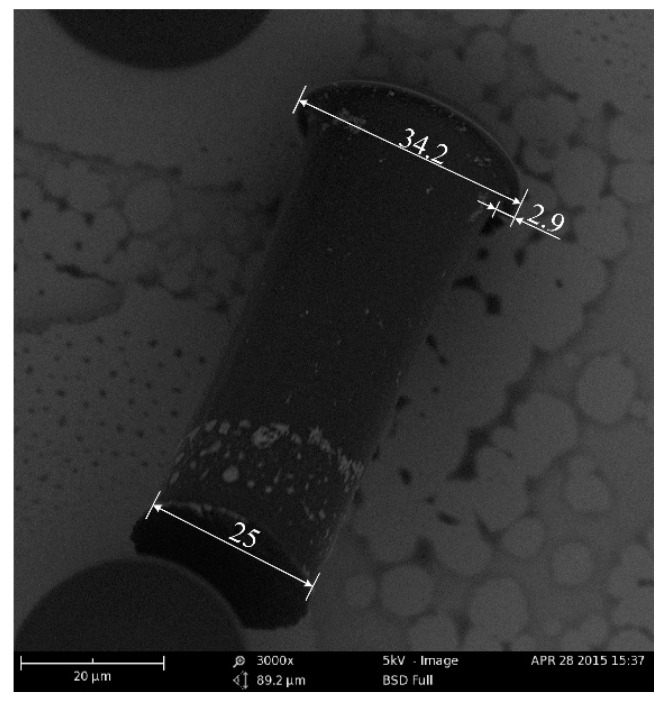
Scanning electron microscope (SEM) image showing the microstructure of the T-topping pillar. The diameter of the top is 34.2 µm, the diameter of the bottom is 25 µm, and the length of the top part of the T-topping is 2.9 μm.

To fabricate the T-topping pillar, a wideband UV source without a filter was used in the exposure process. The microstructure was characterized by a scanning electron microscope (SEM). As shown in Figure 3, the diameter of the top was 34.2 µm, the diameter of the end was 25 µm, and the length of the extended part of the T-topping pillar was 2.9 µm. The geometrical edge angle of the straight wall pillar was 90°. The contact angle of the SU-8 film was tested by a contact angle measuring device. The result showed the contact angle of the SU-8 film was 82°, which is insufficient for the straight wall pillar surface to sustain droplets in the Cassie state. However, the T-topping structure changes the geometrical edge angle of the straight wall pillar from 90° to 0°, enabling the droplet in Cassie state on the prepared surface if the intrinsic water contact angle of the surface is larger than 0°.

Gap and wavelength were the key factors to control the error of the SU-8 structure [49]. The small gap between the photo mask and photoresist reduced the diffraction error. Because the thick SU-8 film was not absolutely flat, controlling the gap homogeneity between photo mask and resist was hard. The other key factor was the exposure wavelength. If the wavelength was shorter than 350 nm, the top region was strongly exposed. The redundant acid would diffuse during the post exposure bake stage, and lead to a T-topping.

### 3.2. Effect of Structural Parameters

Droplet on the T-topping pillar array surface is in Cassie state. Then the apparent contact angle θ_c_ for a suspended droplet is described by the Cassie-Baxter [51] model:

cosθ_c_ = f_s_ (cos θ_y_ + 1) − 1 (1)

where θ_c_ is the apparent contact angle on the textured surface, f_s_ is the fraction of the real liquid-solid contact area to the entire interface, and θ_y_ is the equilibrium contact angle on the smooth surface. 

**Figure 4:**
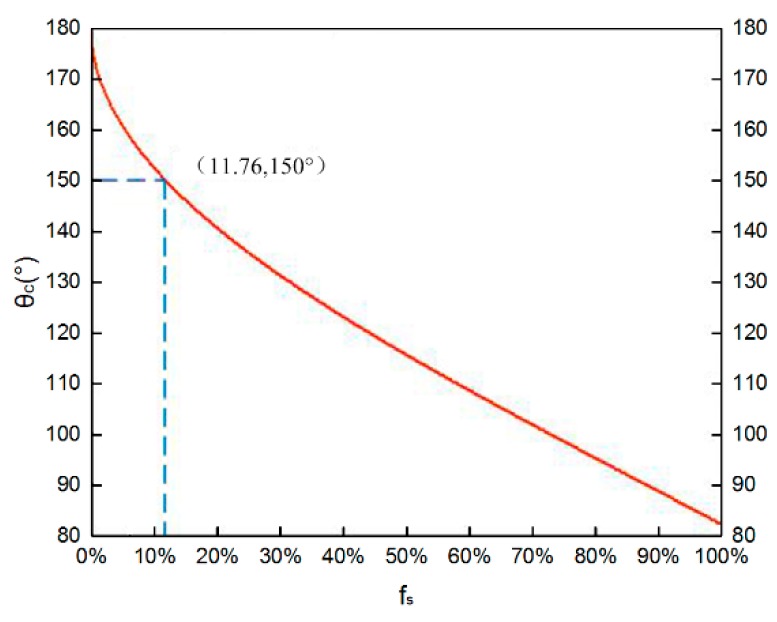
Apparent contact angle (θ_c_) of the textured SU-8 surface as a function of the fraction of the real liquid-solid contact area to the entire interface (f_s_). As f_s_ decreases, the apparent contact angle θ_c_ of the surface increases to 150°. The dashed line indicates the value of f_s_ at θ_c_ = 150°.

The contact angle on the smooth SU-8 film was 82°, so the apparent contact angle θ_c_ is a function of f_s_ according to Equation (1). A typical plot of θ_c_ (Figure 4) shows that as f_s_ decreases, the apparent contact angle of the surface increases. The dashed line indicates the value of f_s_ at θ_c_ = 150°. If f_s_ < 11.76%, θ_c_ is above 150°.

For the structure we designed, considering the water drop contact with N hollowed-out pyramid-like unit-cells, the real liquid-solid contact area is S_r_ = 4Nπ(D/2)^2^ and the entire liquid-solid interface area is S = NP^2^. Then, the fraction of the solid contact with the liquid is

f_s_ = πD^2^/P^2^ (2)

If D/P < 0.19, θ_c_ is above 150° and the surface becomes superhydrophobic. However, with decreasing f_s,_ we wanted to determine if contact angle would increase. According to a previous study [19], if the f_s_ deceases until F_C_ > F_E_ during the entire self-assembly (F_C_ is the capillary force, and F_E_ is the elastic restore force), the micropillars will be unstable. The micro pillars will randomly collapse. 

The parameters of the micro structure influence the self-assembly process. The capillary force must be large enough to bend the pillars against each other. The height and diameter determine the stiffness of the micro pillar. Short and large diameters make the pillars hard to bend. Large and small diameters make the micro pillar easy to bend, which means it is hard for the micro pillars to resist random interference. Hence, proper ratio of height and diameter is very important. The capillary force on an individual pillar in a system of four pillars has been explored [19,23], and was used for structure calculations. Because the capillary force during the self-assembly is complicated and time-varying, providing a precise formula is hard. Previous studies only provided an approximate formula after simplification. According to these studies, we calculated and estimated the structure parameters near the real critical value. For testing, the optimal numerical parameters need more theoretical analysis and experimentation.

The distance of the two adjacent pillars L is important for the stability of self-assembly as it determines the displacement of the top of the pillars. According to previous studies, as the distance between two adjacent pillars decreases, F_C_ increases, and F_E_ decreases. This facilitates self-assembly, and improves the stability of self-assembly.

### 3.3. Surface Morphology

**Figure 5:**
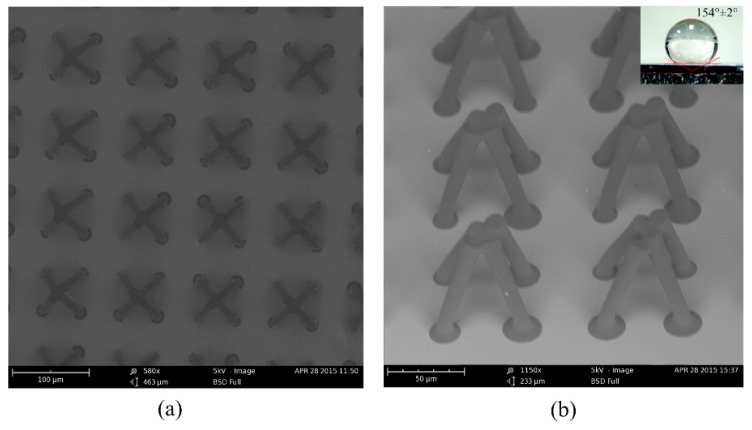
SEM images of the hollowed-out pyramid-like arrays. (**a**) Top view of the hollowed-out pyramid-like arrays; the top of the adjacent pillars are obviously joined together; (**b**) a 45°-tilted view of the hollowed-out pyramid-like surface. (Inset) A droplet of water on top of the prepared surface. The water drop on the surface was about 7 μL, and the static contact angle was 154 ± 2°.

Surface morphology is very important for the wettability of a surface. The microstructures were characterized by SEM. Figure 5a shows the top view of the fabricated microstructures: an array of hollowed-out pyramid-like unit-cells. The unit-cell is composed of four adjacent pillars. The distance between two adjacent periodic unit-cells was 100 µm, and the diameter of the top of the single pillar was about 15 μm, and the diameter of the bottom of the pillar was about 11 µm. Because only the four circles on the top contact the droplet of water, the fraction of the solid in contact with liquid is about 7.07%. Thus, the static contact angle should be larger than 150° according to the Cassie-Baxter model. From Figure 5b, we obtained the whole image of the fabricated superhydrophobic microstructure. The periodic unit-cell was a hollowed-out pyramid structure like the top of the *Salvinia molesta* leaf. The bottom of the pyramid-like structure was square and the side length was 50 µm. Thus, we successfully performed the difficult fabrication process and created the hollowed-out pyramid-like structure. The wettability of the sample was measured. The water drop on the surface was about 7 μL, and the static contact angle was 154 ± 2°. The water drop used to test the sliding angle was about 10 μL, and the sliding angle of the surface was 10 ± 1°.

**Figure 6:**
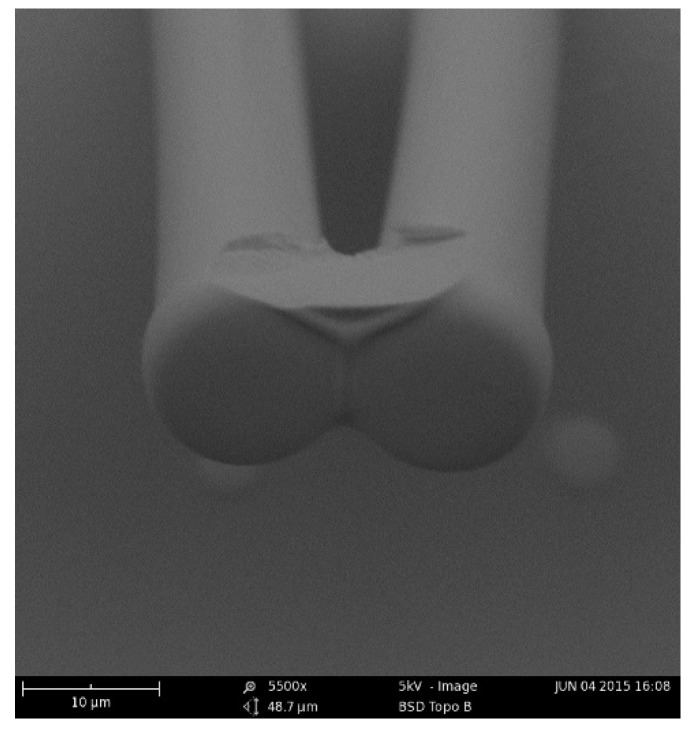
SEM image showing the remaining SU-8 photoresist bonding formation around the micro pillars. Two pillars remain. The fracture of the structure shows the remaining SU-8 photoresist bonds the four adjacent micro pillars together.

Adhesive force is required to bond the microstructures after collapse, which was proven again by our study. Polymer weld was examined to bond the micro pillars [22,52]. Although the liquid bridge could pull the four adjacent micro pillars close together, a strong force is still required to bond the pillars together after the water evaporated completely. As shown in Figure 6, some SU-8 adhered on the pillars during the last process of development; the remaining SU-8 photoresist bonds the four adjacent micro pillars together.

The rate of water evaporation is a key factor for bond formation. We placed the sample in the oven at 50 °C to accelerate the evaporation rate, no bond formed, and the collapsed pillars reverted back to their upright position after complete evaporation. Additionally, when the sample was placed in a closed glass container at 20 °C to slow the evaporation rate, the result was the same, showing that a proper evaporation rate is important for self-assembly. This area requires further research.

For superhydrophobic surfaces, structure defects are unacceptable. A small defect could make the Cassie state of the droplet break down. However, obtaining a defect-free sample is difficult. Many factors affect the self-assembly process, especially the bond strength between the wafer and the micro pillars.

The bond strength between the wafer and the micro pillars is the essential factor that can provide the force that the pillars need to stay standing. If the bond strength is rather small, the micro pillars will fall down, or even flow away during the developing process. Several approaches to achieve adhesion between the wafer and the micro pillars are possible, such as wet etching with the solution mixed by H_2_SO_4_ and H_2_O_2_, cleaning by oxygen plasma, or pre-spin coated with Polymethyl Methacrylate (PMMA). Obviously, the wet etching process can’t be used for metal basement. Damp bottom are extremely harmful for the bond strength, so baking the bottom in the oven for a period of time to maintain dryness is necessary before spin coating the SU-8. 

### 3.4. Environmental Stability

**Figure 7:**
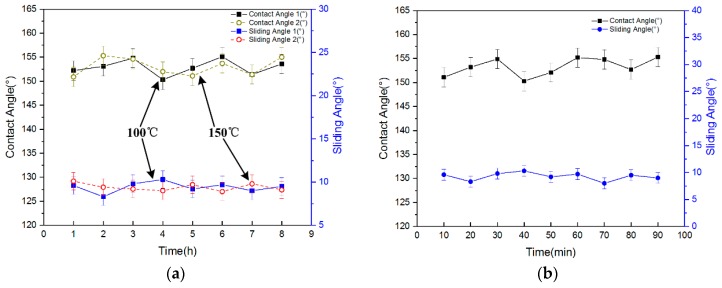
(**a**) The contact angles and sliding angles of the surface after high temperature thermal treatment. Contact angle 1 and sliding angle 1 are the results of the surface after 100 °C thermal treatment, contact angle 2 and sliding angle 2 are the results of surface after 150 °C thermal treatment. (**b**) The contact angle of surface after ultraviolet (UV) irradiation.

The wettability of the sample under severe environmental conditions was tested. To test the high temperature durability of the surface, the sample was placed on a hotplate at 100 °C for one hour. Then the sample was removed from the hotplate and cooled to room temperature. The contact angles were tested, and the sample was placed back on the hotplate at 100 °C for another hour. This process was repeated eight times. The temperature was then raised to 150 °C, and the experiment above was repeated. The results are shown in Figure 7a. The contact angles fluctuated between 150 and 155°, and the sliding angles were about 10°. No obvious difference compared with the surface before the thermal treatment was observed. Because the glass transition temperature (T_g_) of Su-8 is greater than 200 °C, the contact angle and sliding angles will not be affected after heating at lower temperatures. This result confirms that the T-topping features were not altered and the water repellency of the structured surfaces was stable, even when subjected to very high temperatures. The sample showed a high temperature bearing capacity. The surface maintained superhydrophobicity after exposure to high temperatures, so the surface can be applied in high temperature situations.

In the UV durability tests, the sample was irradiated with UV for up to 90 min, and the contact angles of the sample were measured every 10 min. The result is shown in Figure 7b. The contact angles fluctuated between 150 and 155°, and the sliding angles were about 10°. No obvious difference was observed compared with the surface before UV irradiation. The surface maintained superhydrophobicity after UV irradiation for a long time, showing an excellent UV bearing capacity.

**Figure 8:**
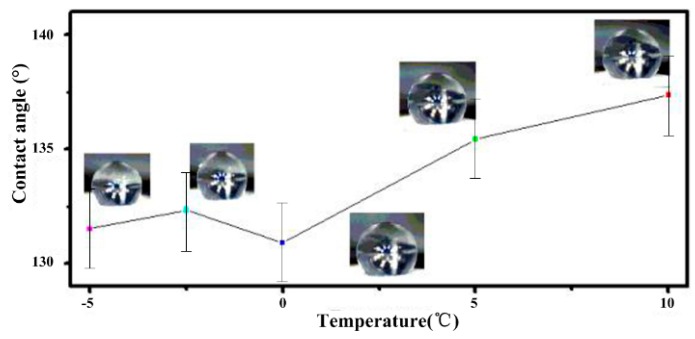
The water contact angles for the prepared surface during the cooling process as a function of temperature. When the temperature decreased from 10 to −5 °C, the static contact angles decreased from 137.5 ± 2° to 131.4 ± 2°.

The relationship between the static contact angle and low temperature was investigated. The sample was cooled on the refrigeration table, and the side view of the water droplet and the corresponding temperature were recorded. As shown in Figure 8, the static contact angles changed slightly when the temperature fell from 10 to −5 °C. Because the cooling process was rapid, the water drop did not freeze. Because the condensation of the water from the air would impact the wettability and cause the switch from Cassie-Baxter to Wenzel, we controlled the humidity of the air using the thermostat. The humidity was 30%. When the temperature fell from 10 to −5 °C, the static contact angles decreased to less than 10°. The surface maintained hydrophobicity when the temperature decreased, so the structure has a strong ability to adapt to falling temperatures.

## 4. Conclusions

Inspired by the micro structure of the *Salvinia molesta* leaf, we designed and fabricated a periodic hollowed-out pyramid microstructure with excellent superhydrophobicity by combining capillary-induced self-assembly and photolithography technology. SU-8 photoresist was used as the structural material in this fabrication. To maintain the droplet on the micropillar array in Cassie state, T-topping pillars were constructed to improve the geometrical edge angle. The evaporation-induced assembly collapsed the pillar and formed the hollowed-out pyramid structure during the structure release step. The water contact angle of the surface was 154 ± 2° and the sliding angle of the surface was 10 ± 1°. Additionally, the prepared surface showed excellent wettability after long exposure to high temperatures and ultraviolet irradiation, and the contact angle of the surface fluctuated slightly when the temperature dropped. 

For the hollowed-out pyramid-like surface, considerable space remained between the micro pillars, and the interspace surrounded by the hollowed-out pyramid-like structures was better for air retention, so the surface has potential to retain air under water. Compared with the previous durable superhydrophobic surfaces [39,40,41,42,43,44,45], the water contact angle of the prepared surface only reached superhydrophobicity, and the sliding angle of the prepared surface must be improved. The durability against mechanical abrasion, chemical stability, and organic solvents need further research. However, the prepared surface attained superhydrophobicity by changing the micro structure of the basement materials without any fluorinated alkyl silane or modified silica nanoparticles. The surface demonstrated stable wettability against high temperatures, cooling, and UV resistance, which are very important for practical application. Additionally, the hollowed-out pyramid-like structures can be fabricated by other materials with good mechanical properties. The surface has the potential for self-cleaning and drag reduction under water. Further research may focus on the wettability of the micro structure array boundary, because this region will inject water to the surface if the surface becomes immersed in water.
